# Water regime and osmotic adjustment under warming conditions on wheat in the Yaqui Valley, Mexico

**DOI:** 10.7717/peerj.7029

**Published:** 2019-06-12

**Authors:** Leandris Argentel-Martínez, Jaime Garatuza-Payan, Enrico A. Yepez, Tulio Arredondo, Sergio de los Santos-Villalobos

**Affiliations:** 1 Instituto Tecnológico de Sonora, Cd. Obregón, Sonora, México; 2 Tecnológico Nacional de México/Instituto Tecnológico del Valle del Yaqui, Bacum, Sonora, Mexico; 3 Instituto Potosino de Investigación Científica y Tecnológica, San Luis Potosí, SLP, México; 4 CONACYT-Instituto Tecnológico de Sonora, Cd. Obregón, Sonora, México

**Keywords:** Water potential, Osmotic potential, Transpiration, Climate change, Osmolytes

## Abstract

An experiment was carried out to evaluate the effect of increased temperature on roots and leaf water and osmotic potential, osmotic adjustment (OA) and transpiration on *Triticum durum* L. (CIRNO C2008 variety) during growth (seedling growth), tillering and heading phenophases. Wheat was sown under field conditions at the Experimental Technology Transfer Center (CETT-910), as a representative wheat crop area from the Yaqui Valley, Sonora México. Thermal radiators were placed at 1.20 m from the crop canopy. Treatments included warmed plots (2 °C) and ambient canopy temperature with five replicates. Temperature treatment was controlled using a (proportional, integrative, derivative) feedback control system on plots covering a circular area of *r* = 1.5 m. Results indicated a significant decrease in the osmotic potential of roots and leaves for the warmed plots. Water potential, under warming treatment, also experienced a significant reduction and a potential gradient was observed in both, roots and leaves, while the phenophases were delayed. Such results demonstrate that, under warmer conditions, plants increase water absorption for cooling. Hence, transpiration experienced a significant increase under warming in all phenophases that was related to the low root and leaf water potential. CIRNO C2008 also experienced OA in all phenophases with glycine betaine as the osmolyte with major contribution.

## Introduction

Plants exposed to high temperatures increase transpiration as a mechanism for foliar cooling to avoid enzyme denaturalization and leaf dehydration, inducing water stress (Turner, 2017). This condition affects cell homeostasis, whereas several biochemical and physiological processes take place to lower the osmotic potential (Martínez‐Vilalta & Garcia‐Forner, 2017) and the plant water potential (Acosta-Motos et al., 2017). In order to maintain homeostasis, plants exhibit mechanisms for adjusting osmotic conditions and changing osmotic pressure inside the cells, a process called osmotic adjustment (OA) (Blum, 2017). Stressed plants decrease osmotic potential by accumulating low molecular weight and osmotically active compounds (osmolytes) such as proline, glycine betaine (GB), and reduced glutathione (RG). Proline and GB have been extensively studied in several crops including wheat (Tian et al., 2017) due to their positive correlation of increased concentration with OA and yield under drought stress. When osmotic potential is low in plants tissues, plants warrant water uptake from the soil (Turner, 2017). In plants capable of carrying out OA, root growth, water absorption, cell elongation, leaf area, photosynthesis, transpiration, and yield are maintained close to its genetic-productive potential (Naeem et al., 2016). Some studies demonstrate that in wheat the OA occurred under salinity (Argentel Martínez et al., 2017; Blum, 2017); however, under warming, this physiological mechanism might also occur to avoid cellular damage. With adequate irrigation conditions in non-saline soils, no adjustment in water relations would be expected. Still, warming might exacerbate transpiration causing OA adjustment to maintain favorable transpiration rates.

The semiarid Yaqui Valley is a highly productive wheat area that requires intensive irrigation to maintain yields (Garatuza-Payán et al., 1998), with CIRNO C2008 the most extensively cropped wheat variety (Figueroa-López et al., 2010). Recent climate change predictions (Cavazos & Arriaga-Ramírez, 2012; Navarro-Estupiñan et al., 2018) point at temperature increases in the following decades. Yield penalties are associated with chronically high temperatures and with heat shocks and, as suggested by Cossani & Reynolds (2012), spring wheat breeding targeted for abiotic stress delivers better genetic gains in warmer environment.

Hence, evaluation of responses of water relations responses to warming have prime importance due to the implications that increased temperatures may have for crop aclimatation (Kimball et al., 2012; Argentel Martínez et al., 2017), likely bringing profound effects on wheat yields as pointed out by modeling synthesis studies (Mendoza-Ochoa et al., 2018). The objective of this study was to evaluate the effects of increased temperature on osmotically active compounds, water and osmotic potentials, OA, and transpiration of the most common wheat variety under field conditions, to understand the variability of water regime as possible indicator of heat stress tolerance under future climate change.

## Material and Methods

The experiment was carried out during the whole growing season of 2016–2017 (December–April), under the field conditions of the Yaqui Valley, Sonora, Mexico, at: 27°22′0.4″N and 109°54′50.6″W (UTM: 607393.24 m E; 3027508.34 m N), 47 masl.

The crop response to increased canopy temperature was studied based on the methods described in Garatuza-Payan et al. (2018) and Kimball (2005), where five plots were subjected to increased canopy temperature, during the whole growing season, by 2 °C (Warming treatment) with respect to ambient canopy temperature (Control treatment) (Kimball, 2011). Each of the five plots of the warming treatment were equipped with six thermal radiators (FTE-1000 model; Mor Electric Company Heating Association Inc. Comstock Park, MI, USA), (Garatuza-Payan et al., 2018, Kimball, 2015) mounted on equilateral triangular structures of 5.2 m by side (two radiators on each side) forming a hexagon. Plots were also equipped with infrared temperature sensors (IRTS Apogee Instruments Inc., Logan, UT, USA) pointing to the center of each plot for temperature control (Garatuza-Payan et al., 2018). Temperature differences of 2 °C between warming and control plots were maintained throughout the routine described in Kimball (2015).

The CIRNO C2008 wheat variety was used as experimental model due to its high biomass and grain yield stability since 2008, reason why it is still widely used in Northwest Mexico (Figueroa-López et al., 2010). Field agricultural practices were described in Garatuza-Payan et al. (2018). Seeding was carried out with a sowing machine (SUB-24) on December 8th, 2016 on a vertisol soil with three rows on the furrows and a seed density of 170 kg ha^−1^. Fertilization was applied at seeding, using 250 kg ha^−1^ of Urea and 100 kg ha^−1^ of monoammonium phosphate (MAP) fertilizer, 11-52-00. Additional fertilization was applied during the first and second irrigation at a dose of 50 kg ha^−1^ of urea. This occurred when gravimetric water volume of the soil was about 70% at an average water depth of 14 cm.

For water potential (Ψ_*L*_) measurements, 24 randomized samples of five seedlings (leaves *a* and *b*, that is, the two most exposed leaves to sunlight) per plot were taken at both, 5:00 (pre-dawn) and at 10.30 am to determine the current water potential. Right after collection, the samples were dissected into its organs (roots and leaves) and water potential was measured with a Schollander pressure bomb (PMS-100; PMS Instrument Company, Albany, OR, USA). During measurements, samples were quickly placed into double-zip bags and sealed. Roots and leaves were then placed in the chamber sample holder and pressure was applied until sap was observed in the exposed cut.

Criteria to classify water stress in plants was determined based on Ψ_*L*_ values, and was defined as: non-stress when Ψ_*L*_ > −1.0 MPa, moderate stress between −1.0 > Ψ_*L*_ > −1.4 MPa, and severe when water potential dropped Ψ_*L*_ < −1.4 MPa (Ruiz-Sánchez et al., 2017).

For osmotic potential (Ψ_*s*_), measurements occurred under saturated weight conditions, thus 24 roots and leaf samples per treatment were taken and placed in Petri dishes for rehydration with distilled water, which were introduced in double-closing ziploc bags and were maintained at 8 °C for 12 h. The samples were then wrapped with aluminum foil for freezing in liquid nitrogen and stored in a freezer at −80 °C. Samples were afterward thawed at room temperature and centrifuged at 3,000 rpm during 3 min and root and leaves cell juice was obtained. Ψ_*s*_ was determined from aliquots of 100 mL with a vapor pressure osmometer (Vapro 7120; ELITechGroup, Smithfield, RI, USA).

Osmotic adjustment was calculated as the difference of the saturated osmotic potential of leaves between control and warming treatments (Turner, 2017).

Proline concentration was determined as described by Bates, Waldern & Teare (1973) and Rienth et al. (2014) based on a standard curve and calculated on a fresh weight basis as follows: ((μg proline mL^−1^ toluene)/115.5 μg μmole^−1^)/((g sample)/5) = μmoles proline g^−1^ of fresh weight. GB was extracted following Grieve & Grattan (1983) and expressed on μmoles GB/g of fresh weight. RG was quantified following the spectrophotometric technique established by Xue et al. (2001) in a UV–VIS spectrophotometer at 412 nm. The values were expressed in nmol g FW^−1^.

Transpiration (E in mmol H_2_O m^−2^ s^−1^) in leaves *a* and *b* was measured in both warming and control treatments in three phenophases, using a LI-6400XT system (Portable Photosynthesis System, Li-Cor, Lincoln, NE, USA). Transpiration was measured at a saturated photon flux density of at least 1,500 μmol m^−2^ s^−1^. Measurements were done during initial growth (seedling growth), tillering and heading phenophases. All transpiration, water and osmotic potential measurements were carried out the same day.

For each response variable, assumption of normality and homogeneity were tested and means and its standard deviation and standard error were determined. Differences between means were detected by a *t*-student test. For OA, proline, GB, and RG comparisons between phenophases, an analysis of variance was carried out based in a linear model of fix effects and differences were detected by a post-hoc Tukey test comparison for significance level at 1%. Determination coefficient (*R*^2^) without adjustment was determined to evaluate phenophases contribution to general variability. To determine osmolyte contribution to OA a regression analysis was carried out taking OA and measured osmolytes as dependent and independent variables, respectively. Correlation coefficient (*r*) and its significance were determined for each equation. For all statistical analysis professional software STATISTICA was used.

## Results and Discussion

**Figure 1:**
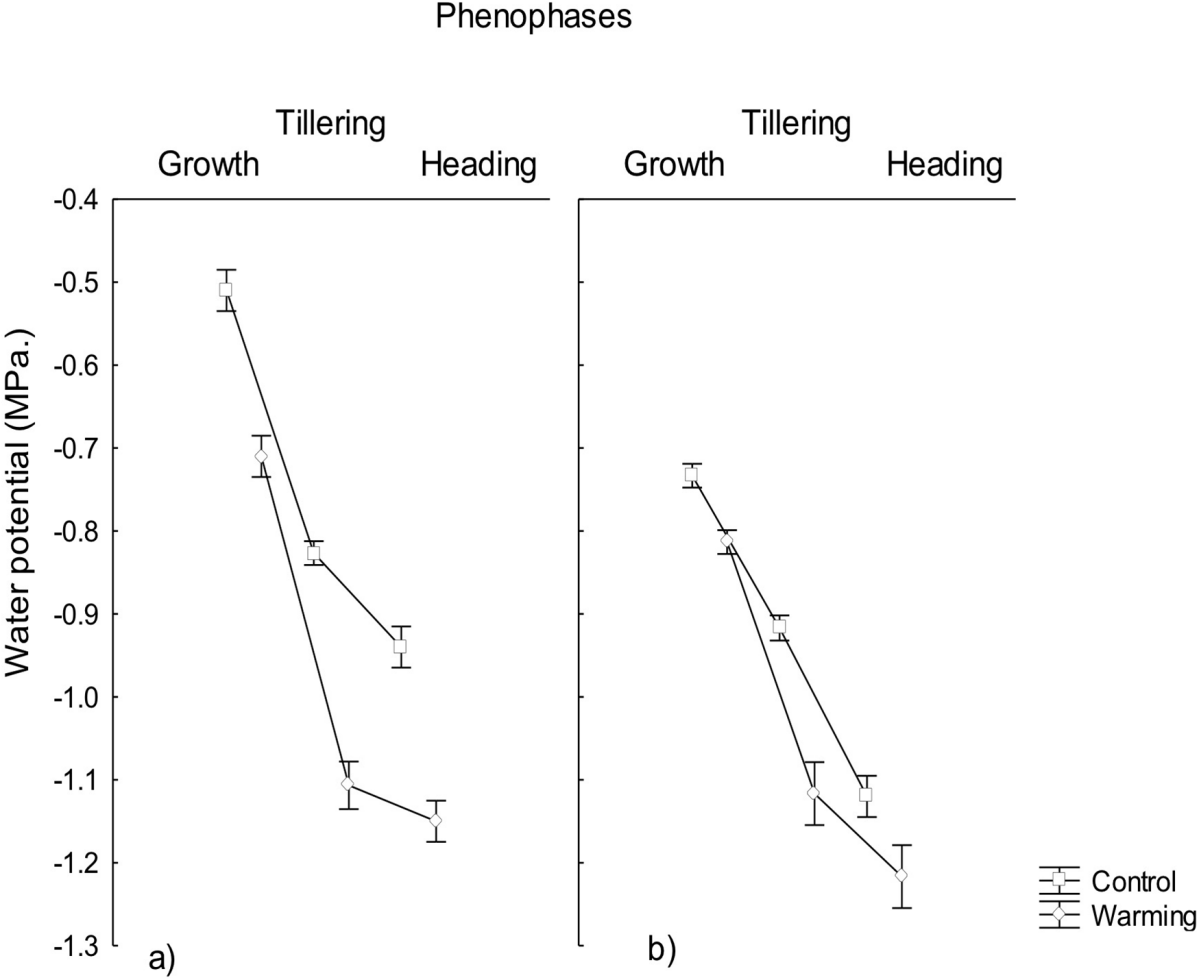
Water potential in roost and leaves in different phenophases. Water potential in (A) roots and (B) leaves of CIRNO C2008 during Growth, Tillering and Heading under Warming and Control treatments. Rectangular bars represent the standard deviation from the means.

Water potential was significantly lowered under the warming treatment in the three phenophases evaluated, observing a water potential gradient going from the roots to the leaves (Fig. 1) likely to warrant a correct water status in plants for transpiration avoiding foliar damage and enzymes denaturalization under warmer conditions. The observed minimum water potential values were −1.1 MPa in roots and −1.2 MPa in leaves, during heading. Based on the Ψ_*L*_ values the only phenophase considered under moderate stress was heading in leaves (i.e., Ψ_*L*_ < 1.2 MPa). Given that the irrigation was the same across treatments, the difference found in the water potentials was probably due to the effect of the imposed warming conditions and not to differences in the moisture content of the soil.

Water potential reduction in plants allows absorbing a sufficient amount of water in order to maintain adequate transpiration and to avoid mesophyll damage under heat. This phenomena has been observed in wheat under salinity (Sadras et al., 2016) and drought stress (Saxena et al., 2016) but there is not much information about water and osmotic potential reductions under heat stress. In the present study, warming conditions generated stress during the heading phenophase, suggesting that in order to overcome physiological stress induced by warming, plants responded with a significant increase of osmotically active compounds to decrease its osmotic potential and increase water uptake (Tasmina et al., 2017).

**Figure 2:**
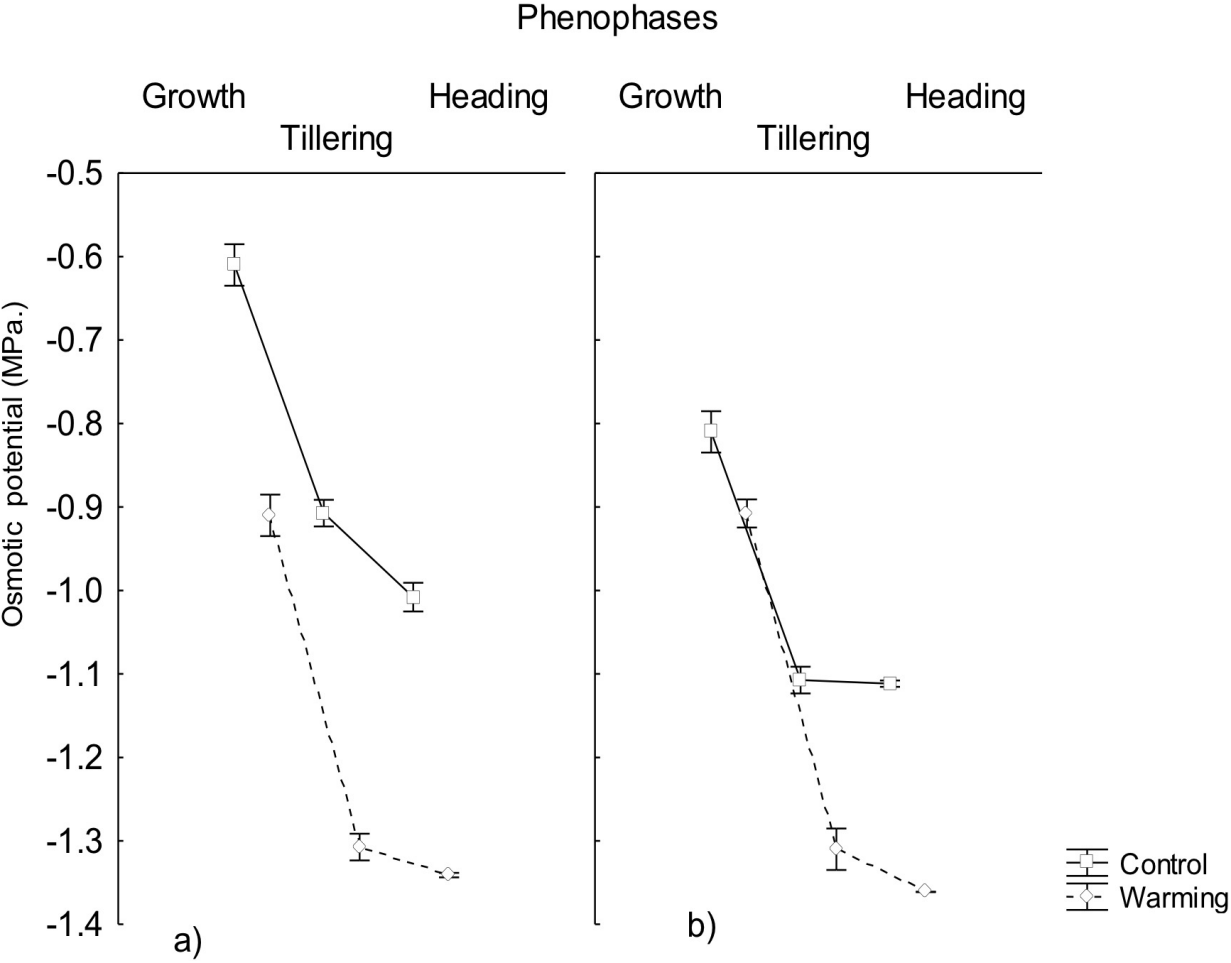
Osmotic potential in roots and leaves in different phenophases. Osmotic potential in (A) roots and (B) leaves of CIRNO C2008 variety during Growth, Tillering and Heading under Warming and Control treatments. Bars represent the standard deviation from the means.

In accordance, the study showed that the osmotic potential also decreased significantly in all phenophases under warming and it was always lower than the water potential (Fig. 2). This result is explained by the high accumulation of osmotically active compounds synthesized during the exposition to warming and/or perhaps due to mechanisms of salt inclusion (data not determined) which also contributes to osmotic pressure increase and, consequently, to a decrease of the osmotic potential, mostly in roots.

The capability to decrease osmotic potential in plants, as the one recorded in the present assay, has been an important response variable to consider for breeding programs because of their role to mitigate physiological drought effects (Dell Amico et al., 2016). This response will allow to maintain an adequate water balance under heat conditions when transpiration increases for mesophyll cooling and to avoid foliar damage and mesophyll warming under such conditions.

**Figure 3:**
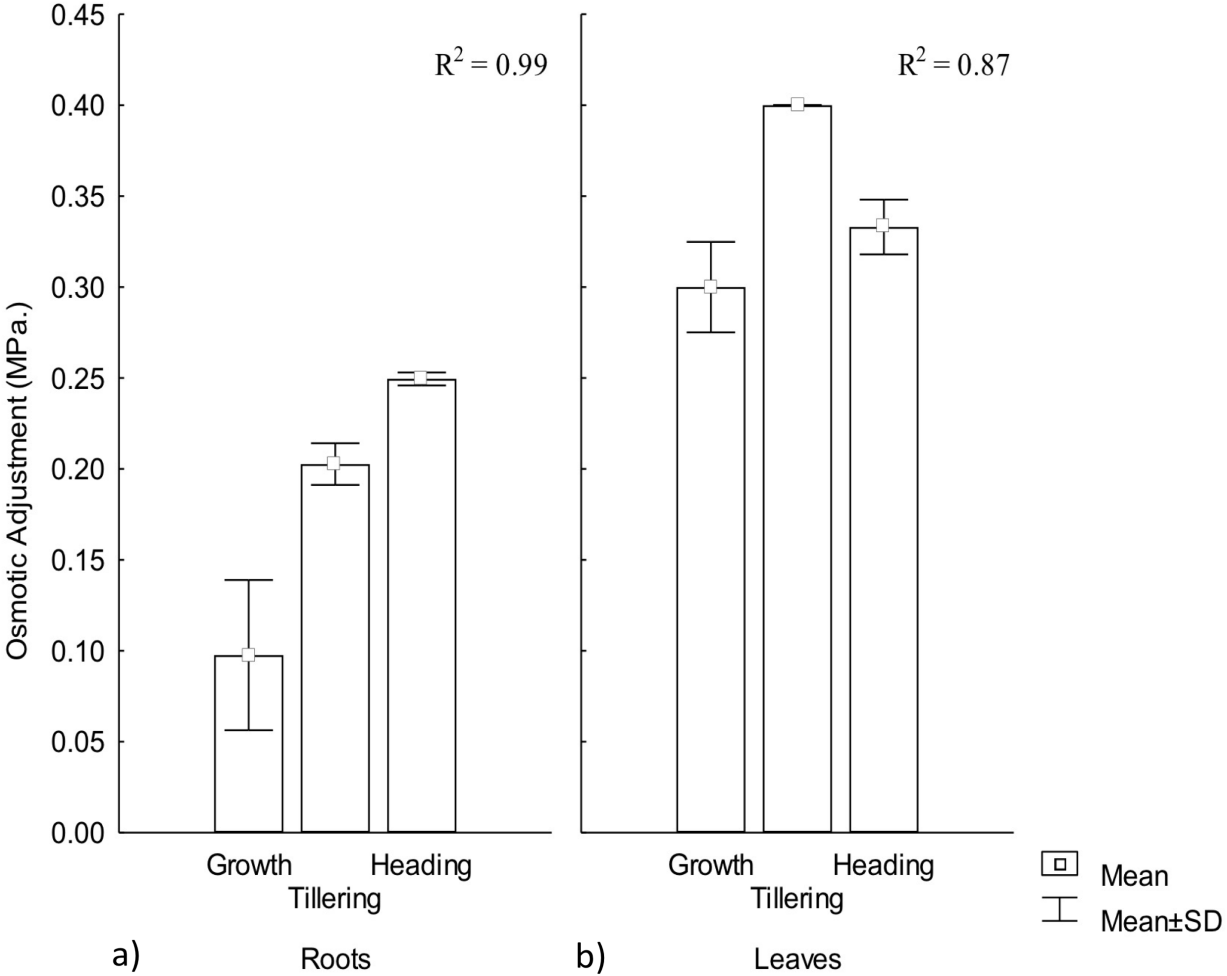
Osmotic adjustment in roots and leaves in different phenophases. Osmotic adjustment in (A) roots and (B) leaves during Growth, Tillering and Heading phenophases. *R*^2^: Determination coefficient without adjustment. Rectangular bars represent the standard deviation from the means.

Osmotic adjustment plays an important role for plant adaptation to low water availability (Turner, 2017), mainly through the maintenance of cell turgor and the protection of cellular functions (Blum, 2017). OA was observed in both roots and leaves, demonstrating a significant effect by warming conditions, particularly among phenophases. In roots the highest adjustment was observed during heading but in leaves it occurred during tillering. Thus, OA variation in CIRNO C2008 was attributed in 99% and 87%, for roots and leaves, respectively, due to the warming treatment along phenophases. On the other hand, leaves showed the highest OA adjustment, demonstrating the existence of an OA gradient in plants that contribute to a favorable water regime (Fig. 3).

Osmotic adjustment has been proposed as a selection variable for genetic improvement in rice (*Oriza sativa* L.) and maize (*Zea maiz* L.) under warming conditions due to its close water-yield relationship (Turner, 2017). For wheat improvement, OA variable has recently been included in different models for combined abiotic stress tolerance (Zandalinas et al., 2017), mainly for semi-arid climates where crop water availability is less than 80% of crop’s water demand. OA is positively related to yield under water stress (Thapa, Wick & Chatterjee, 2017). When OA under water stress reaches up to 0.1 MPa, higher photosynthesis and yield has been observed (Jajoo & Allakhverdiev, 2017).

**Figure 4:**
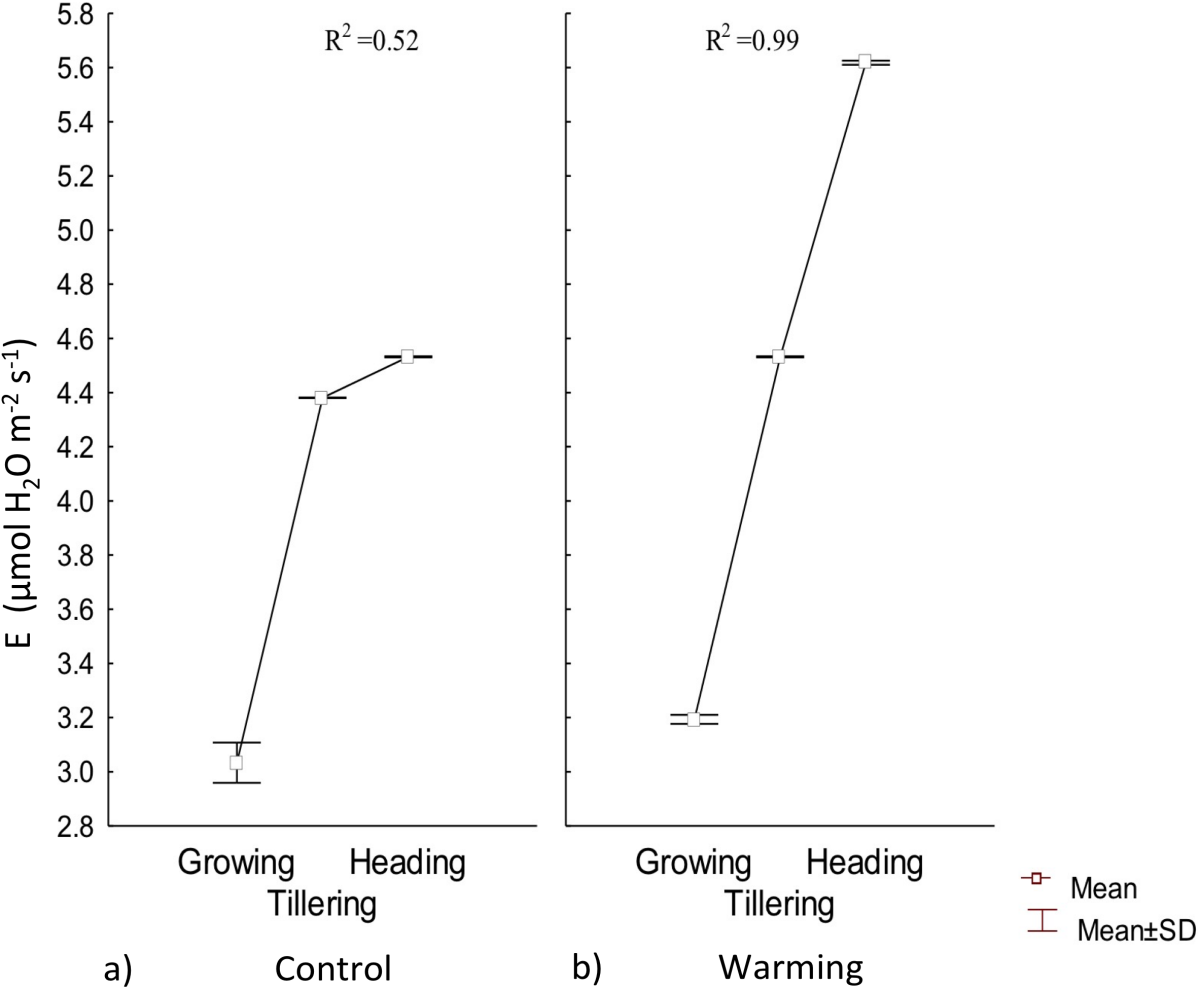
Transpiration in different phenophases. Transpiration during Growth, Tillering and Heading under (A) Control and (B) Warming. Rectangular bars represent the standard deviation from the means.

Transpiration experienced a significative increase under warming during the tillering and heading phenophases; however, during the first stage of growth there were no differences with respect to the control (Fig. 4). Also, under Warming, phenology explained 99% of the transpiration rate increase. In contrast, phenology only explained 52% of transpiration variability in the control.

Water availability in the soil, atmospheric vapor pressure, and temperature are the most important factors in determining transpiration (Martínez‐Vilalta & Garcia‐Forner, 2017). Under warmer temperatures, transpiration is more intense but less stable over time because of a rapid partial or total stomatal closure to avoid mesophyll dehydration (Sharma, 2016). In the present assay, high transpiration rates were probably accelerated for thermoregulation during tillering and heading.

Some studies confirm that partial or total stomatal closure is not a good adaptive response to thermal stress, since stomatal closure reduces water loss, causing a temperature increase at both apoplastic and symplastic level and, subsequently, cell damage (Perdomo et al., 2015). Plants solve temperature damages increasing transpiration. This process, like many others involved in thermal stress responses, is also regulated by abscisic acid (Mohammadi, 2012). Stomatal closure can, as well, affect photosynthesis rate because of the CO_2_ fixation reduction (Khatun, Ahmed & Mohi-Ud-Din, 2015).

**Figure 5:**
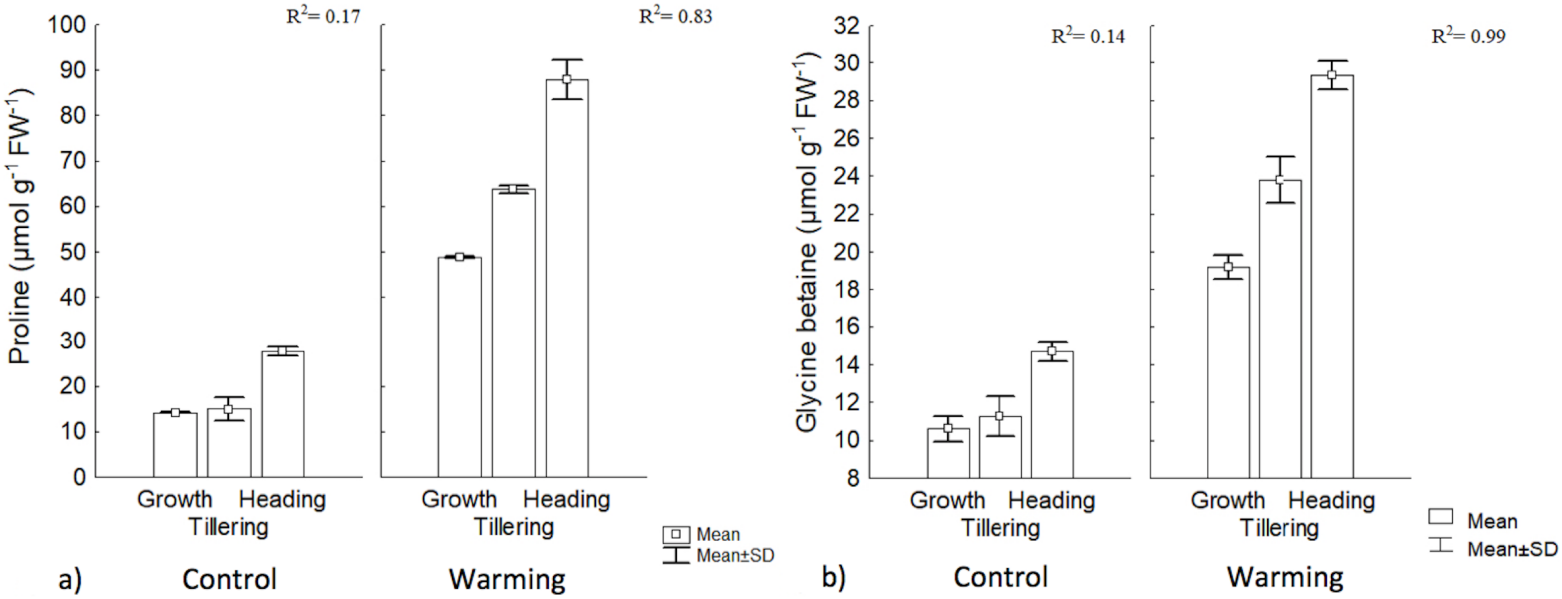
Proline and glycine betaine contents in different phenophases. (A) Proline and (B) glycine betaine contents during Growth, Tillering and Heading phenophases. *R*^2^: Determination coefficient without adjustment. Rectangular bars represent the standard deviation from the means.

Proline and GB content increased significantly under warming in the three phenophases. The highest value for GB was obtained during heading, although its concentration was lower than proline. In the control, there were no differences, between the first two phenophases, but concentration differed with respect to heading. With warming, phenology explained 83% and 99% of proline and GB contents increases, respectively, while no changes in such osmolytes were observed in the control (Fig. 5).

The increase observed in proline content with warming could be an important signal of heat tolerance by CIRNO C2008 since proline participates in multiple processes that prevent cell damage in plants, such as a mediator of OA in several species (Tavakoli, Poustini & Alizadeh, 2016). Proline is also a stabilizing metabolite of proteins and membranes (Çoban & Baydar, 2016). Proline can also be used in plants as a carbon and nitrogen source, readily available during cell rehydration (Signorelli, 2016) and as a source of reduction equivalents (proline catabolism) to support oxidative phosphorylation and Adenosine Triphosphate (ATP) regeneration during recovery to stress. In cereals under stress, proline also helps to control cytosol acidosis and can maintain NADH_2_/NAD^+^ relationships at values compatible with metabolism in several crops including wheat (Saibi et al., 2016). A significant increase of proline and GB during heat stress exposure, for tillering, has been found by some authors (Rienth et al., 2014), and both proline and GB content were increased with increased temperature. Such authors also found that proline and GB were correlated in at 58% and 66%, respectively, with a heat tolerance index.

Among the compatible solutes, GB is a particularly effective protector during drought mitigating the detrimental effects of oxidative stress by activating or stabilizing enzymes that capture reactive oxygen species (Blum, 2017; Liu, Lin & Huang, 2017). Although not all wheat varieties show significant increases in GB (i.e., more than 150 μmol g^−1^ FW^−1^) during drought (Saxena et al., 2016), CIRNO C2008 increased its concentration in all phenophases under warming, suggesting that this osmolyte is also an effective biochemical protection against heat. In this respect, Tian et al. (2017) reported that in wheat under warming stress, GB content increased to 120 μmol g^−1^ DW^−1^, and even when heat and water stress were combined, GB content increased to 150 μmol g^−1^ DW^−1^. Increased accumulation of compatible intracellular solutes, such as proline and GB, results in an increased cellular osmolarity that favors water inflow and reduces outflow, maintaining turgidity under stress conditions (Wilson et al., 2014).

Equally, RG is a non-protein tripeptide that contributes to decrease the osmotic potential in plants by increasing solute concentration and the subsequent water potential reduction favoring water absorption to fulfill the transpiratory demand (Nahar et al., 2015).

**Figure 6:**
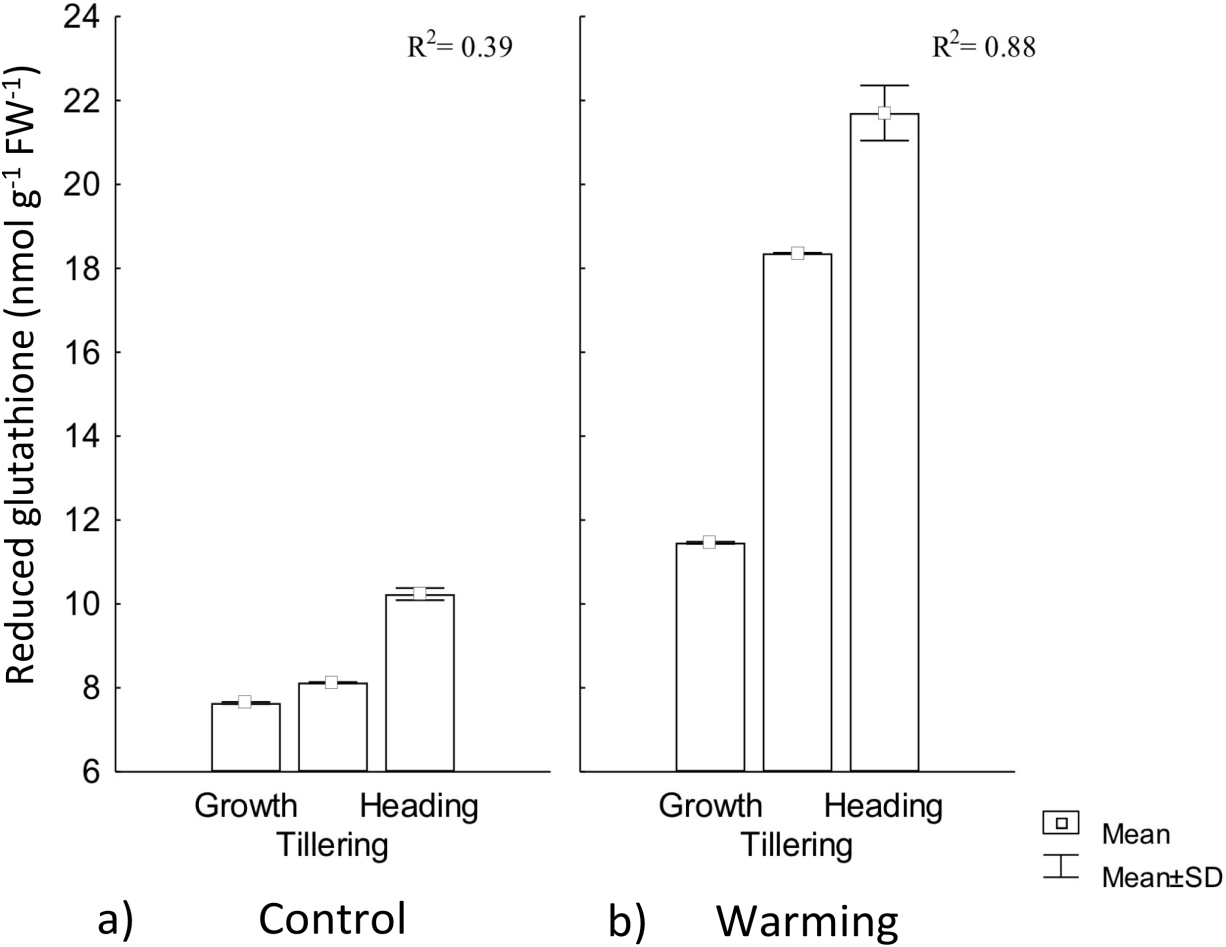
Reduced glutathione foliar content in different phenophases. Reduced glutathione foliar content during Growth, Tillering and Heading phenophases under (A) Control and (B) Warming. *R*^2^: Determination coefficient without adjustment. Rectangular bars represent the standard deviation from the means.

In our study, RG content experienced a significant increase by warming during all phenophases. In this treatment, phenology explained 88% of RG increase, whereas for the control, phenology showed no regulation on RG (Fig. 6). This response to warming is likely related to the need of absorbing additional water (lowering water potential) to compensate the high transpiration demand for leaf cooling.

In response to drought stress, most hydrolyzed enzymes increase their activity, and some proteins are degraded to low molecular weight precursors in order to increase osmolytes concentration that are then hydrolyzed to tripeptides and dipeptides (Ohama et al., 2017). In our study, RG increased content demonstrates CIRNO C2008’s capability to tolerate heat driven by its accumulation for OA (Zandalinas et al., 2017).

Several reports stated that the effect of abiotic stress varies according to the degree of varieties tolerance (Mohammadi, 2012; Ramani et al., 2017), where some genotypes have shown different responses when exposed to the same stress condition, but an effective way to alleviate the stress intensity is achieved by increasing low density or low molecular weight proteins concentration such as RG (Khan et al., 2017). This increase occurs due to the high RG synthase enzyme activity, whose catalytic action is dependent on ATP and NAPH_2_ generally coming from the photochemical phase of the photosynthetic process, an aspect that may affect plants productivity during its acclimatization and tolerance to conditions of warming stress (Tao et al., 2015).

Analyzing osmolyte concentration vs. OA relationship, the metabolite that most contribute to OA variability was GB (*r* = 0.84), followed by proline and RG, which also showed a significant correlation (Table 1).

**Table 1:** Correlation between osmotic adjustment and osmolytes.

Relationship	Regression equation	*r* value	*R*^2^
OA-PRO	OA = 0.14 + 0.003*PRO	0.83*	0.67
OA-GB	OA = 0.05 + 0.012*GB	0.84*	0.71
OA-RG	OA= 0.06 + 0.015*RG	0.82*	0.66

**Notes:**

Correlation between osmotic adjustment and proline, glycine betaine, and reduced glutathione during Growth, Tillering and Heading phenophases.

* Significance for *p* < 0.05.

Although it is known that osmotically active metabolites in plants play particular roles under normal conditions, when stress conditions arise, the biochemical signal of the OA is activated, leading to the degradation of high molecular weight biomolecules to its monomeric bases for the OA (Blum, 2017). The analyzed osmolytes in the present study are probably an example of the activation of this mechanism during plant stress response.

Our research is the first assay attending the warming effect on the water regime of wheat at the Yaqui Valley under field conditions, and revealed the capacity of CIRNO C2008, used as an experimental model, to decrease osmotic potential for water adjustment and water uptake under increased temperature. These responses suggest the possibility for using these characteristics as indicators in breeding programs to adapt to warming climate scenarios.

## Conclusions

Warming imposed to CIRNO C2008 led to significant modifications in the water relations of wheat, showing a decrease in water and osmotic potentials during growth, tillering and heading phenophases. There was a water potential gradient between root and leaf for normal symplastic water status maintenance along phenophases. Variations in water potential caused by temperature induced the occurrence of a significative OA. GB content was the osmolyte of greater contribution to OA under warming.

Also, the maintenance of the water potential gradient and its low values promoted high transpiration levels during tillering and heading, indicating the capability of CIRNO C2008 to tolerate physiological drought associated to high transpiration under temperature increase in crop canopy.

The capacity of CIRNO C2008 for osmotic potential adjustment and water uptake under increased temperature was revealed, indicating the possibility for using these variables as reference indicators in breeding programs for climate change scenarios.

## Supplemental Information

10.7717/peerj.7029/supp-1 Supplemental Information 1 Water regime database. Water and osmotic potential, osmotic adjustment, transpiration and Proline, GB and RG contents raw data.

## Additional Information and Declarations

### Data Availability

The following information was supplied regarding data availability: The raw data is available in the Supplemental File. It shows water and osmotic potential, osmotic adjustment, transpiration and proline and reduced glutathione content, in different phenophases.
